# Development of an instructor guide tool: ‘Three Stages of Holistic Debriefing’

**DOI:** 10.1590/1518-8345.3089.3229

**Published:** 2020-02-03

**Authors:** Fernanda dos Santos Nogueira de Góes, Deirdre Jackman

**Affiliations:** 1 Universidade de São Paulo, Escola de Enfermagem de Ribeirão Preto, PAHO/WHO Collaborating Centre for Nursing Research Development, Ribeirão Preto, São Paulo, Brazil.; 2 University of Alberta, Faculty of Nursing, Edmonton, Alberta, Canadá.

**Keywords:** Faculty, Nursing, High Fidelity Simulation Training, Education Nursing, Education, Higher, Learning, Teaching, Docentes de Enfermagem, Treinamento com Simulação de Alta Fidelidade, Educação em Enfermagem, Educação Superior, Aprendizagem, Ensino, Docentes de Enfermería, Enseñanza Mediante Simulación de Alta Fidelidad, Educación en Enfermería, Educación Superior, Aprendizaje, Enseñanza

## Abstract

**Objective:**

to describe the development of an English and Brazilian Portuguese Holistic Debriefing Tool focused on nursing educator to promote a reflective learning.

**Method:**

a methodology study, with three phases: integrative literature review; tool development and review of a panel of nursing experts. The literature review tracked a systematic process. For the tool development were used literature review results, Lederman’s Debriefing Process and Zabala’s learning framework as theoretical referential to promote a reflective learning in High-Fidelity Simulation. The panel of nursing experts analysed the quality of the tool.

**Results:**

literature review evidenced gaps about educator pedagogical preparation and indicated no holistic debriefing tool exists which captures formative and summative aspects of debriefing guidance to assist the educator to debrief. Debriefing tool was purposed with two pages: first page were recommended how conduct debriefing and second page is a questions guidance. The tool evaluation was undertaken for a total of three modifications for congruence and concept reader clarity.

**Conclusion:**

it was proposed a holistic debriefing tool focused on nursing educator. This study provides an overall picture of the process to promote a reflexive learning in High-Fidelity Simulation and to contribute to formal nursing educator training to apply best pedagogical practice.

## Abstract (pt)

**Objetivo:**

descrever o desenvolvimento de um guia holístico de *debriefing*, em inglês e português do Brasil, focado em enfermeiros educadores para promover a aprendizagem reflexiva.

**Método:**

estudo metodológico, com três fases: revisão integrativa de literatura, desenvolvimento do guia e sua avaliação por um painel de especialistas em enfermagem. A revisão de literatura seguiu um processo sistemático. Para o desenvolvimento do guia, os resultados da revisão de literatura foram utilizados, juntamente com o processo de *debriefing* de Lederman e o referencial de aprendizagem de Zabala, como base teórica para promover a aprendizagem reflexiva durante a Simulação de Alta Fidelidade. O painel de especialistas em enfermagem analisou a qualidade do guia.

**Resultados:**

a revisão da literatura revelou lacunas na preparação dos educadores pedagógicos e a ausência de ferramentas holísticas de *debriefing* que incluam os aspectos formativos e sumativos de orientação em *debriefing* para auxiliar os educadores. O guia de *debriefing* foi desenvolvido em duas páginas: a primeira página recomenda como conduzir o *debriefing* e a segunda contém perguntas de orientação. O guia foi avaliado e passou por um total de três modificações para congruência dos itens avaliados e adequação dos termos utilizados.

**Conclusão:**

foi proposto um guia holístico de *debriefing* visando enfermeiros educadores. Este estudo fornece uma visão geral do processo para promover a aprendizagem reflexiva na Simulação de Alta Fidelidade e contribui para o treinamento formal de enfermeiros educadores para a aplicação das melhores práticas pedagógicas.

## Abstract (es)

**Objetivo:**

describir el desarrollo de un Instrumento del *Debriefing* Holístico en inglés y portugués brasileño dirigido al educador de enfermería para promover un aprendizaje reflexivo.

**Método:**

estudio metodológico, con tres fases: revisión integradora de la literatura, desarrollo del instrumento, y revisión de un panel de expertos en enfermería. La revisión de la literatura siguió un proceso sistemático. Para el desarrollo del instrumento se utilizaron los resultados de la revisión de la literatura, el proceso de *debriefing* de Lederman y el sistema de aprendizaje de Zabala como referencial teórico para promover un aprendizaje reflexivo en la Simulación de Alta Fidelidad. El panel de expertos en enfermería analizó la calidad del instrumento.

**Resultados:**

la revisión de la literatura evidenció carencias en la preparación pedagógica del educador e indicó que no existe un instrumento integral de *debriefing* estructurada que capture los aspectos formativos y sumativos de la orientación del *debriefing* estructurado para ayudar al educador a realizarlo. El instrumento de del *debriefing* estructurado se preparó utilizando dos páginas: la primera muestra cómo realizar del *debriefing* estructurado y la segunda es una guía de preguntas. La evaluación del instrumento se realizó para un total de tres modificaciones para la congruencia y la claridad de lectura de los conceptos.

**Conclusión:**

se propone un Instrumento del *Debriefing* Holístico dirigido al educador de enfermería. Este estudio proporciona una visión general del proceso para promover un aprendizaje reflexivo en la Simulación de Alta Fidelidad y contribuir a la capacitación formal del educador de enfermería para aplicar la mejor práctica pedagógica.

## Introduction

In the last decade, High Fidelity Simulation (HFS) has been used extensively in nursing education as a resource for teaching and learning. HFS is defined as learning activities which can replicate the practice setting in order to achieve specific educational goals and attend to the social requirements of maintaining patient safety^(^ 1 ^)^.

Debriefing is an essential component of all simulation experiences^(^ 1 ^-^ 4 ^)^. It is defined as a pedagogical method whereby students are guided by a facilitator through a reflective thinking process thus assisting them to connect theory to practice and to understand concepts within the simulation scenario^(^ 1 ^,^ 3 ^)^. 

Debriefing has a number of benefits to nursing education including reflective thinking process thus assisting nursing students to connect theory to practice^(^ 5 ^)^; an important step to engaged students in meaningful learning^(^ 6 ^-^ 7 ^)^; to support students in deconstructing the learning activity and then synthesize the experience to reinforce the learning activity for future recollection^(^ 8 ^)^; to facilitate experiential learning to develop/hone skills, to reduce negative feelings and to connect the simulated activities to real-life clinical situations^(^ 2 ^,^ 5 ^-^ 6 ^)^. 

Without this reflection *(debriefing)* stage, the effectiveness of the simulation activity can be greatly diminished and hinder the students’ assessment of the activity and its connection to previously built learning while in their programs of study^(^ 2 ^,^ 5 ^-^ 7 ^,^ 9 ^)^. 

As a central and basic component of HFS debriefing is universally accepted; however, is unclear how nurse educators are taught to apply best pedagogical practice^(^ 9 ^-^ 10 ^)^. Despite the indicated value of debriefing to the learning process, particularly in nursing education, research remains poorly articulated^(^ 2 ^,^ 9 ^)^. There is a lack of evidence in relation to the ability of the educator/instructor/debriefer to support, to guide, to observe, to evaluate and to direct actions, discussions and reflections of the student during the debriefing component of HFS. 

It is imperative for the nurse educator to be knowledgeable in how to conduct debriefing, including consideration of best pedagogical practice^(^ 8 ^-^ 9 ^)^. The question arises that for nurse educators, does utilization of a Debriefing Tool serve to provide relevant information to apply best pedagogical practice^(^ 11 ^)^ in HFS, wich means the nurse educator has competency to facilitate learning, to brief supportive and immersive feedback and to promote effective communication? 

Although some debriefing assessment tools^(^ 12 ^)^ reflect core components of debriefing, they focus specifically on student perspective. Thus, there is a lack of consensus and an absence in the literature in relation to formalized resources targeting debriefing in simulation to assist the nurse educator to conduct debriefing which considers the best pedagogical practice and becomes an important ally for the development of nursing students’ clinical acumen^(^ 7 ^,^ 13 ^-^ 14 ^)^. 

Considering that simulation is utilized globally and the lack of best pedagogy is universal, the purpose of this paper is to describe the development of an English and Brazilian Portuguese Holistic Debriefing Tool focused on nursing educator to promote a reflective learning. 

Therefore this tool in more than one language accommodates the usage of the tool in a global manner to be shared in many institutions with transferred applicability.

## Method

A methodological study^(^ 15 ^)^ for the development of an English and Brazilian Portuguese Debriefing tool focused on nursing educator to promote a learning guide aimed to help the nurse educator/instructor to promote best pedagogic practice in relation to simulation debriefing. 

This study followed three phases: the first one was an extensive integrative literature review to support the development of the tool; the second was the tool development *per si*; the last phase was the de submission of the tool to the judgment of a panel of nursing educator/ research experts who conduct/teach and lead HFS and debriefing. 

In the first phase authors conducted an integrative literature review of the issues related to the field of the study. The research question guiding the review was as follows ‘how does the educator/debriefer conduct HFS debriefing with graduate/undergraduate nursing students, including consideration of best pedagogical practice’? 

Data base sources included Virtual Health Library (VHL), CINAHL, Scopus, PubMed and Web of Science using keywords to Web of Science and Scopus, descriptors to PUBMED, and combination of them to VHL and CINAHL. The terms used were “high-fidelity simulation”, debriefing, “education, nursing”, “students, nursing” or “undergraduate nursing” or “graduate nursing student”, “case studies”, “health case”, and “clinical case”. 

The criteria inclusion applied for this review were: articles who about debriefing process; HFS and debriefing process rolled with nursing students (undergraduate and graduate); articles published in English, Spanish or Portuguese, between 2005-2016. The exclusion criteria were: not primary studies, editorials, and studies not developed with nursing students and faculty. Data collection was performed between September and October 2016; searches in all databases occurred in one week in the beginning of September, 2016. 

The literature review contributed with evidence to support the tool development to guide nurse educator/instructor to promote best pedagogic practice in relation to simulation debriefing. The results reinforce what developers chosen about debriefing’ definition, methods and technics; name to be given for who conduct debriefing process and the relevance to include attitudinal, procedural and cognitive learning.

In the second phase the tool was developed in accordance with the literature review findings, the theoretical referential of debriefing of Lederman (1992)^(^ 16 ^)^ and attitudinal, procedural and cognitive learning process of Zabala (1998)^(^ 17 ^)^. 

The framework of Lederman’s Debriefing Process argues that debriefing is composed by three steps: “the systematic reflection and analysis (participants systematic self-reflective process about the experience through which they have just come”); “intensification and personalization (refocusing of participant’s reflections onto their own individual experience and the meanings they have for them)”; and, “generalization and application (participants from their own individual experience to the broader applications and implications of that experience)”^(^ 16 ^)^. 

The framework of Zabala (1998) sustains the learning process could occur in attitudinal, procedural and cognitive perspective, which are extrinsically connected, to one another. In addition, tool developers’ approached with author proposes of grouping and join in teaching-learning activities or sequences of instruction, relations and communicative situations between teachers and students in order to socially and organize the group, distribute space and time^(^ 17 ^)^. 

Depending on the focus and student educational levels of knowledge, the nurse educator must customize the simulation experience to entail one aspect of a learning modality, or the simulation may be leveled in complexity to include higher-level knowledge and skill application, which targets cognitive, attitudinal and procedural learning simultaneously^(^ 16 ^-^ 17 ^)^. 

Data collection was between November 2016 to March 2017; draft number 10 was considered adequate to the expert’s revision.

Finally, the developers relied on their expertise, literature review findings, Lederman’s and Zabala’s frameworks^(^ 16 ^-^ 17 ^)^ to create the tool tool ‘*Three Stages of Holistic Debriefing*’. 

In the third phase, an evaluation of ‘*Three Stages of Holistic Debriefing*’ tool with a panel of five nursing educator/research experts who conduct/teach and lead HFS and debriefing (03 Canadians e 02 Brazilians) was carried out after being informed about the research and giving their written consent . For select the experts^(^ 18 ^)^, a minimum score of five points was adopted according with PhD in Health Science (one point), expertise in HFS Nursing Education (one point), being Nurse Faculty (one point), participate in HFS research group/project (one point) and authorship or co-authorship of paper published in journals about Nursing Education or HFS (one point).

The assessments were forwarded to the experts electronically in April 2017. They were asked to analyze the layout and content of the guide looking for clarity, appearance. This process was undertaken 3 times, until all the experts agreed totally. 

The research was registered in the Brazil/CONEP Platform (CAAE 67357517.8.0000.5393) and approved by the Research Ethics Committee of the Ribeirão Preto Nursing College (2.111.736), according with 466/12 Brazilian Resolution of the National Health Council^(^ 19 ^)^. 

## Results

At the literature review (phase 1), the first search resulted in 220 studies: 61 from Web of Science, 31 from VHL, 44 from PUBMED, 30 from Cinahl and 54 from SCOPUS, which was its abstract and/or full-text was read to analyze the attending of criteria inclusion; 31 primary studies were included which it is related to the debriefing process.

This study identifies while it is primarily faculty members who conduct debriefing, formal pedagogical preparation criteria is absent. Only one study focused on the role of the nurse educator in HFS debriefing including the promotion of best pedagogy. A study indicated that formative debriefing was conducted. Of the total, 16 studies indicated using tools (23 - 12 studies indicated to use more than one tool) to guide or evaluate the debriefing process on student perspective, none of which was developed specifically to assist the nurse educator to conduct debriefing, including attending to particular learning processes such as attitudinal, technical and cognitive modalities. 

As results of the second phase, considering developers’ expertise as nursing educator/research who conduct/teach and lead HFS and debriefing, a framework is being purposing of holistic debriefing including formative and summative debriefing. Tool allows the nurse educator to guide the entire group (hand on HFS experience and group observation) to self-reflection and group reflection through all the simulation experience. Additionally it can also help the nursing faculty to improve their role as educators to conduct the debriefing incorporating pedagogical elements in its performance, through the use of a tool.

According with INACSL Standards of Best Practice^(^ 20 ^)^, the HFS simulation process may occur with small groups to facilitate the formative evaluation, no more than 5 students per educator. For the purpose of this tool, authors define the formative debriefing as a reflection activity that must be performed by the nursing educator during the whole simulation process, considering that debriefing should be a continuous process of reflective thinking for nursing student learning and future decision-making. 

**Figure 1:**
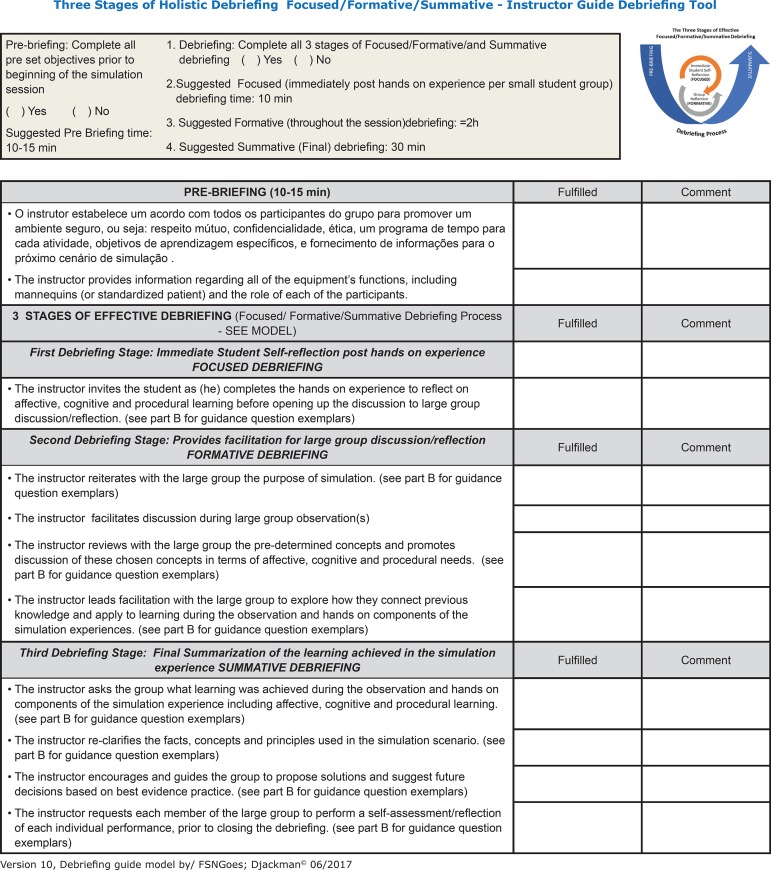
Front page of The ‘Three Stages of Holistic Debriefing’, an instructor guide debriefing tool. From Authors, version ten

This study is purposing a tool called ‘*Three Stages of Holistic Debriefing*’ within three stages, and suggested time for to complete each simulation stage, including the pre-briefing (Figure 1).

- Pre-briefing (Suggested time: 10-15 minutes): the educator may complete all pre-set objectives prior to beginning of the simulation session including establishes a contract with all of the group participants to promote a safe environment:
- mutual respect, confidentiality, ethics, a scheduled outline of the time allocated for each activity; specific learning objectives, the role of each participants;
- provision of information for the upcoming simulation scenario, provides information regarding all of the equipment’s functions, including mannequins (or standardized patient).
- First Debriefing Stage: *Immediate Student Self-reflection post hands on experience/focused debriefing. (*Suggested time: 10 minutes per student/group). The educator:
  to invites the students immediately post hands on experience to reflect on affective, cognitive;
  procedural learning before opening up the discussion to large group discussion/reflection. This stage must be repeat immediately post hands on experience per small student group.
- Second Debriefing Stage*: Provides facilitation for large group discussion/reflection/formative debriefing* (Suggested time: 2 hours) While the student/group goes to their hands on simulation experience, the educator stimulates the entire group (who are in the observation room) to discuss and thinking critically throughout the simulation session:
  the instructor reiterates with the large group the purpose of simulation;
  the instructor facilitates discussion during large group observation(s);
  the instructor reviews with the large group the pre-determined concepts and promotes discussion of these chosen concepts in terms of affective, cognitive and procedural needs;
  the instructor leads facilitation with the large group to explore how they connect previous knowledge and apply to learning during the observation and hands on components of the simulation experiences.
- Third Debriefing Stage*: Final Summarization of the learning achieved in the simulation/ experience - summative debriefing* (Suggested time: 30 minutes): This is the final stage and must be done after all the group have had their hand on simulation experience:
  the instructor asks the group what learning was achieved during the observation and hands on components of the simulation experience including affective, cognitive and procedural learning;
  the instructor re-clarifies the facts, concepts and principles used in the simulation scenario;
  the instructor encourages and guides the group to propose solutions and suggest future decisions based on best evidence practice;
  the instructor requests each member of the large group to perform a self-assessment/reflection of each individual performance, prior to closing the debriefing.

**Figure 2:**
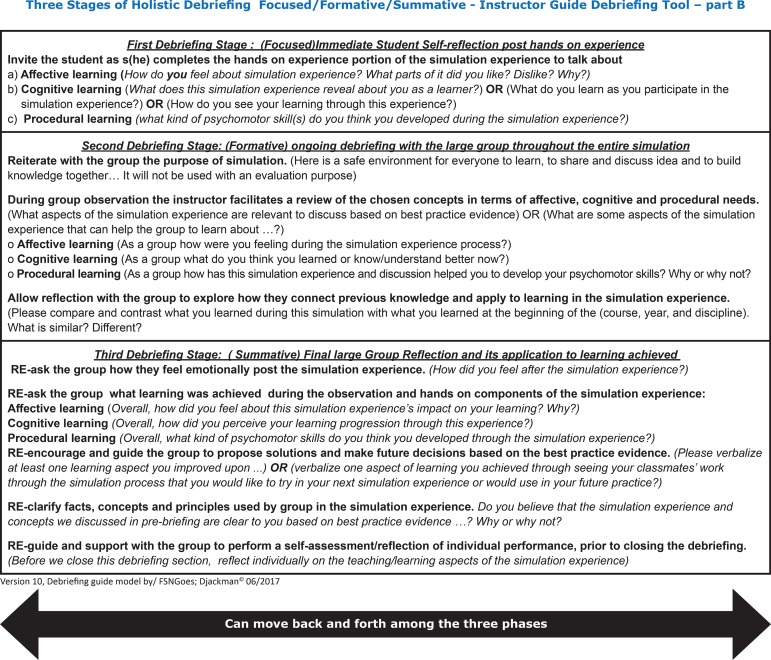
Back page of ‘*Three Stages of Holistic Debriefing’, guidance question exemplars – part B*. From Authors, version ten

Additionally, on the back of the sheet were included a guidance question exemplars for each orientation in each stage to facilitate for novice educator to conduct a debriefing focused on the best pedagogic practice (Figure 2). 

At the third phase, this study conducted an evaluation of tool with five nursing educator/research experts who conduct/teach and lead HFS and debriefing (03 Canadians e 02 Brazilians) invited by authors’ convenience to guarantee the participation of experts. All of them has more than 6 years as educators and use HFS in nursing education; although one of them do not conduct research with HSF/debriefing, she participates in research group/project and authorship or co-authorship of paper published in journals.

This process was undertaken for a total of three modifications for congruence and concept reader clarity. For each version analyzed by experts, the author discussed the suggestions and modify, if necessary. The version 7 (the first one sent to experts) showed an important picture of how the tool is comprehend by nurse educators and helped to improve it. The second and third version (8 and 9) was more focused on image clarification.

## Discussion

Considering that facilitators should be adequately trained in debrief^(^ 21 ^)^, this study presented the development of an English and Brazilian Portuguese Holistic Debriefing Tool focused on nursing educator to promote a reflective learning with consideration to the role of nurse educator in promoting best pedagogic practice and its recommendations for future nursing education. As no other national and international tools were found that focused on the nursing educator, the findings cannot be discussed through comparison, thus being an innovative study.

Within the first phase an extensive integrative literature review, this study investigated the body of HFS debriefing research over a 12-year period, with studies originating from North and South America, Europe and Asia which provides an overall picture of the state of scientific publications related to debriefing as a resource and promotes best pedagogy in HFS nursing education. 

This review provided results highlighting areas where gaps such as there is a lack of studies focused on the role of the nurse educator in the HFS debriefing process where promotion of best pedagogy is attended to or named and there are no defined tools which can specifically assist the nurse educator to conduct debriefing, where attention to the various learning models of attitudinal, technical and cognitive are outlined. 

For the development of the ‘*Three Stages of Holistic Debriefing’* tool, Lederman’s debriefing theoretical referential have been chosen because his theoretical referential claims facilitator may help students to learn and its implication and not to tell them (students) what they may learn. Also, Lederman^(^ 16 ^)^ argues that “in the educational context, the goal is to facilitate an understanding of what has happened, to find out what the participant learned, and to test that against the instructor’s learning objective”. This referential has been used in a range group of scientists in diverse areas^(^ 22 ^-^ 23 ^)^. 

Because in the nursing education its important linking attitudinal, procedural and cognitive learning this study also used Zabala (1998) theoretical referential about learning process^(^ 17 ^)^. These complex activities stimulate a process of personal elaboration of the knowledge. Experimental and practice activities allow new learning content to relate to previous knowledge. Understanding is part of a student’s knowledge not only when he is able to repeat his definition, but also to use it for the interpretation, understanding or exposition of a phenomenon or situation, capable of situating concrete facts, objects or situations^(^ 17 ^)^.

In addition, Zabala (1998) states that best pedagogical practice should align and be responsive to the social, needs of various populations. Pedagogy should also attend to the current and contextual needs of the student including student diversity and autonomy as this influences how students’ process and construct knowledge^(^ 17 ^)^.

Because student must be active during the learning, this study is purposing a formative debriefing. The expression “formative evaluation” dates from 1960s^(^ 24 ^)^ to provides student’s individual feedback of each stage of their learning process.

Debriefing as formative assessment is a highly interactive process in which skills and understanding are not simply dispassionately assessed by the instructor, but in which new insights are co-created in a dialogue between instructor and students^(^ 13 ^)^. 

From author’s teaching practice reinforced by scientific evidence, debriefing only at the end of the whole of the simulation may disinterest students and its influences in learning achievement. Stimulate the student actively participating in the pedagogical proposal improves higher levels of retention when trainees actively think about, analyze, and discuss what happened^(^ 5 ^,^ 23 ^)^. 

The summative assessment occurs at the end of the training session, provides implicit feedback on where the student stands and may prompt changes in the students’ knowledge or behavior, especially through the process of studying for the exam. Formative assessment, occurs throughout the training period and is tailored to the individual learner^(^ 13 ^,^ 25 ^)^, helps develop professional identity through the social interaction of learning conversations and helps to improve clinical skills or teamwork^(^ 25 ^)^. 

Although in this study the authors believe the in-simulation debriefing should be conducted throughout the simulation experience as a way to stimulate the active participation of the student^(^ 5 ^)^ (hand on student and observation group), the tool proposal also allows the debriefer to perform the debriefing at the end of the simulation.

Depending on the physical structure and resources of the simulation center and the availability of safe and ethical spaces for the conduction of the debriefing, it is not possible to carry out the formative debriefing; students must feel they can externalize their knowledge and feelings without being judged and punish by colleagues or nursing educators^(^ 22 ^,^ 26 ^)^. 

The developers’ expertise and discussion in tandem with the external reviewers comments assisted in improving the debriefing tool in terms of clarity, providing a visually comprehensive model and paying due attention to the specific text. The multiple draft revisions sought to create a tool, which had easy utilization for all educators/instructors using HFS in their courses/programs.

Although the Holistic Debriefing Tool seems to be the first one which focus on nursing educator, the absence of others similar tools influences in the possibility to compare the application in nursing education to promotes reflective learning. 

Finally, the construction of educational instruments for nursing education and research foments evidence-based practice, the advancement of scientific knowledge from the pedagogical and methodological framework that guarantees the content validity of the material^(^ 27 ^)^.

For the use of the tool, this study advises:

- Pre-briefing is an important step; it is part of simulation process and helps the student to feel comfortable with the experience.
- Guideline for formative debriefing. The educator must analyses what resources is available at simulation center to choose what the best way to conduct debriefing focused on best pedagogic practice.
- In situations where the formative debriefing is possible, the instructor may give voice for all students immediately after their hand on experience and simulates who does not feel comfortable to reflect loudly their experience.
- The guidance question exemplars are not mandatory; its could help especially novice educators.
- Reinforce with the group the importance of self-assessment and group reflection.

The limitations of the study are on the importance of carrying out prospective studies to follow the application of the tool with Brazilian and English-speaking nurse educator during the planning and execution of the HSF in nursing education to promote the best pedagogical practice for the training of competent and committed nurses with the global health.

## Conclusion

In this methodological study, the authors proposed a new *‘Three Stages of Holistic Debriefing’* tool focused on nursing educator to promote a reflective learning. This study provides an overall picture of development process as a resource, which promotes best pedagogy in HFS nursing education. 

This study presents all the phases to develop an inedited tool including extensive integrative review, use of theoretical referential to supports the development and panel of expert’s analysis. The authors purposed a formative way to conduct debriefing through the whole simulation process, in order to improve higher levels of student’s retention to think critically about, analyze, and discuss what happened. 

Implications for nursing education: the development of ‘*Three Stages of Holistic Debriefing’*, could contributes to formal nursing educator training to apply best pedagogical practice; the tool can also help nursing educators to organize their pedagogical work because it will have a guide reflection helping students to deal with feelings and then with the cognitive issues. This tool can also be used to train nurses educators to use active methodologies and those who wish to include HFS in their pedagogical practice. Pedagogical managers should pay attention to this tool as an auxiliary resource to improve teaching practices in simulation labs. 
